# Role of EMT in drug resistance of breast cancer: molecular mechanisms and therapeutic strategies

**DOI:** 10.3389/fonc.2025.1680751

**Published:** 2025-10-23

**Authors:** Yifan Luo, Renwang Sheng, Xin Tan, Jun Gu

**Affiliations:** ^1^ School of Medicine, Southeast University, Nanjing, China; ^2^ State Key Laboratory of Bioelectronics, School of Biological Science and Medical Engineering, Southeast University, Nanjing, China

**Keywords:** EMT, drug resistance, breast cancer, cancer stem cells, microRNA

## Abstract

Breast cancer, as the most common cancer in women, is a highly heterogeneous and complex tumor. One of the important reasons for the poor prognosis and high mortality of breast cancer patients is drug resistance. More and more evidence shows that epithelial-to-mesenchymal transition (EMT) is a key driver of malignant behavior of breast cancer, and also the core promoter of drug resistance. Multiple EMT-related signaling pathways activate EMT-transcription factors (EMT-TFs) and interact with each other, ultimately inducing drug resistance. The role of EMT in promoting invasion and metastasis has been studied in detail and systematically summarized, but its role in drug resistance of breast cancer has not been elucidated comprehensively. The purpose of this review is to clarify the EMT-related regulatory network in breast cancer and the possible mechanisms of EMT-induced drug resistance. Moreover, we have discussed the potential therapeutic advantages of reversing EMT and drug resistance by effectively targeting key elements of the regulatory network, with particular emphasis on EMT-related signaling pathways and microRNAs. This review summarizes the drug resistance of breast cancer induced by EMT systematically, which is of great significance for solving the drug resistance problem of breast cancer and improving the prognosis of patients.

## 1 Introduction

In recent years, breast cancer, surpassing lung cancer, has become the most common cancer among women and the primary cause of cancer death (1). Breast cancer causes 685,000 deaths annually among cancer patients, far exceeding the data from 2018, which indicates that the burden of breast cancer incidence and mortality is increasing rapidly worldwide (2). Breast cancer is highly heterogenous and has various subtypes, thus it is destined to be a complicated disease to treat, especially triple negative breast cancer (TNBC) which lacks the expression of estrogen receptor (ER), progesterone receptor (PR), and human epidermal growth factor receptor 2 (HER2) (3). According to the guideline, effective therapeutic methods currently used in clinic include chemotherapy, endocrine therapy, targeted therapy, and so on (4). Although tumors are initially sensitive to the anti-tumor drugs, they may develop resistance through various pathways with the continued use of these drugs (5). Widely utilized chemotherapy agents like doxorubicin and paclitaxel combat cancer through their cytotoxic actions. Although TNBC is initially sensitive to chemotherapy than subtypes, most patients gradually develop drug resistance with continued drug administration (6). Tamoxifen is a common endocrine therapy, which can control the progression of ER-positive breast cancer. However, about half of patients with advanced ER-positive breast cancer and almost all patients with metastatic disease have no response to first-line tamoxifen treatment, in addition (7). Trastuzumab, another commonly used drug, has an effective response rate of only 26% as a single first-line treatment in patients with HER2-positive metastatic breast carcinomas (8). Statistical data shows that over ninety percent of cancer deaths are attributed to drug resistance (9, 10). It has always been a persistent factor restricting the therapeutic effect of breast cancer patients (11). Therefore, it is of far-reaching significance to explore the mechanism and treatment strategy of drug resistance in breast cancer.

Drug resistance is frequently complicated and multifactorial owing to the dynamic tumor microenvironment (9). Various intertwined mechanisms interact with each other and signal pathways interfere, ultimately leading to drug resistance (5, 9, 11). In recent years, the important role of epithelial-to-mesenchymal transition (EMT) in drug resistance has gradually been discovered. EMT is a cellular program that involves the transition of cells from epithelial phenotype to mesenchymal phenotype, accompanied by the acquisition of migration ability (12). EMT plays a role in various physiological and pathological processes, including tumor malignant progression and drug resistance (13). Although most of the reports in the past decades have focused on the crucial role of EMT in breast cancer metastasis, increasing evidence shows that EMT is an important mechanism leading to an increased likelihood of drug resistance in breast cancer populations (14). According to the clinical analysis between the chemotherapy response of breast cancer patients and the gene expression profiles of tumor samples, the expression activation of EMT related genes is strongly related to the occurrence of treatment resistance (15, 16). Moreover, Inayatullah et al. used MAST test to compare gene expression profiles and found that chemotherapy resistance in TNBC is related to the activation of EMT program at the beginning of treatment (17). Many studies have reported that the activation of EMT in breast cancer cell lines makes them unresponsive or less sensitive to the treatment of tamoxifen, paclitaxel and doxorubicin (18–20). However, the relationship between EMT and the metastasis of breast cancer has been widely discussed, but the drug resistance caused by EMT has rarely been systematically and completely sorted out, specifically for breast cancer. This review aims to fill this gap and concludes with a discussion on the relationship between EMT and drug resistance, as well as therapeutic strategies targeting EMT for breast cancer.

## 2 EMT: an overview

**Figure 1:**
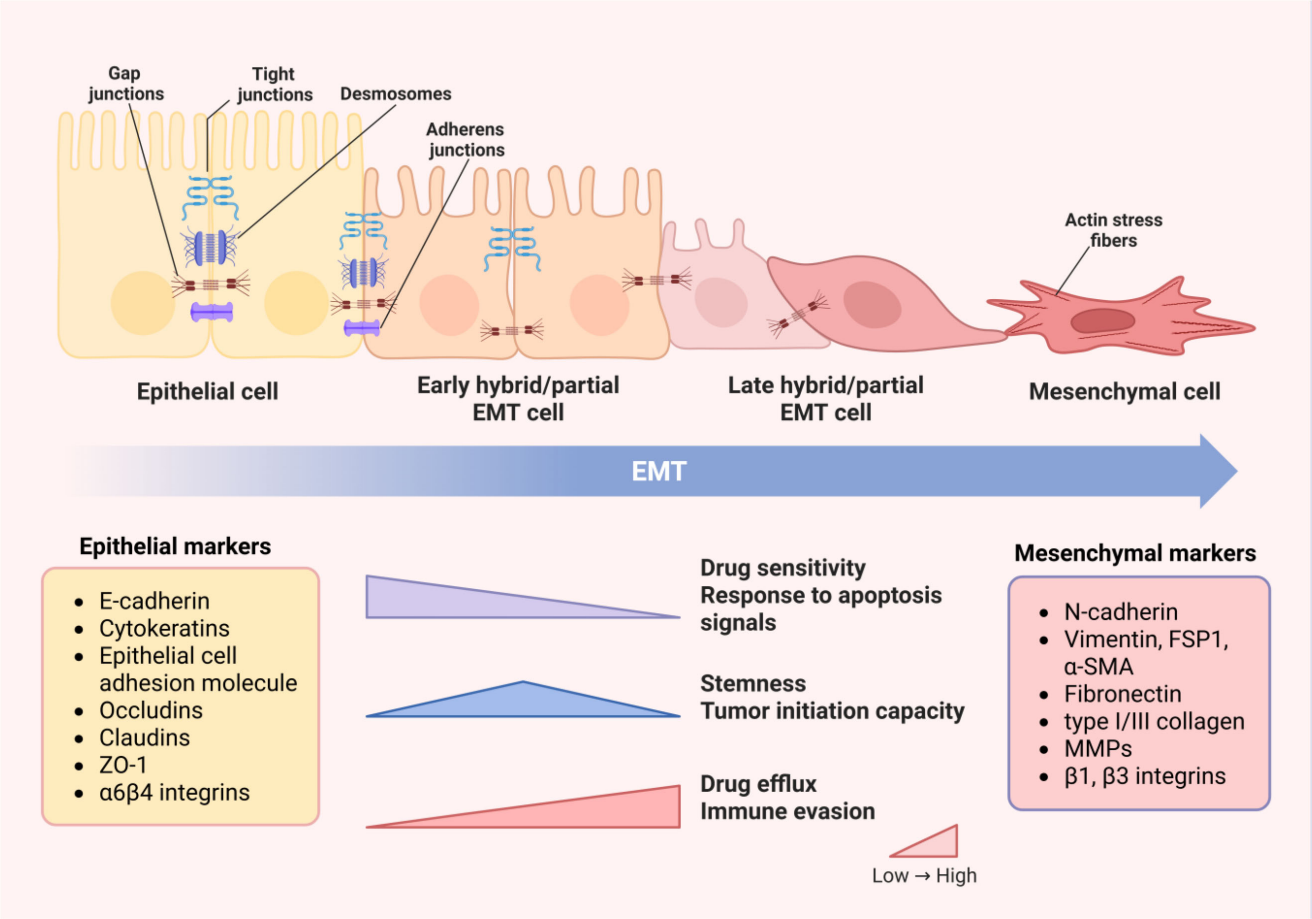
Changes in cell phenotypes and properties during EMT process. With the occurrence of EMT, intercellular junctions dissolve and cell polarity is lost, leading to cytoskeleton remodeling. Therefore, the transformation of cell morphology from epithelial to mesenchymal phenotype and changes in protein expression can be observed. Epithelial markers, including E-cadherin, cytokines, epithelial cell adhesion molecules and tight junction proteins, gradually decrease in expression levels or become inactive, while mesenchymal markers, such as N-cadherin, mesenchymal related cytoskeletal markers, ECM proteins and MMPs, are significantly upregulated. The changes in cell structure and phenotype determine the alterations in properties and functions. The drug sensitivity and response to apoptosis signals of cells decccteristics of drug efflux and immune evasion become increasingly prominent. Cells in the partial EMT state have the highest levels of stemness and tumor initiation ability. Image created using Bio-Render.com software.

EMT is a cellular process that allows polarized epithelial cells to exhibit a mesenchymal phenotype by undergoing various biochemical changes, accompanied with the loss of cell-cell adhesive properties and apical–basal polarity, as well as the acquisition of mesenchymal characteristics, including upregulation of vimentin and loss of cell adhesion (21). (**Figure 1**) During the EMT process, it can be observed that the use of intermediate filaments shifts from cytokeratin to vimentin, and cells achieve higher mobility and invasiveness (12, 22). Cancer cells undergoing EMT can be identified by detecting changes in the expression of EMT-related genes or proteins (23, 24). In the past few decades, research related to EMT markers has been continuously emerging, but the most closely related to the phenotypic changes of EMT are still the loss of cell-cell adhesion and its associated protein expression abnormalities (23). E-cadherin, as a calcium-dependent cell-cell adhesion molecule, is currently the most convincing marker for assessing EMT status, which is also used in clinical diagnosis of cancer progression (24). The downregulation of epithelial marker E-cadherin, accompanied by simultaneous upregulation of mesenchymal marker N-cadherin, is established as a hallmark of EMT. Furthermore, the tight junction components, such as claudins, occludins, zonula occlusions‐1(ZO-1), exhibit downregulation of expression or weakened function when EMT occurs (23, 25). Apart from loss of cell-cell adhesion, change in motility and invasiveness is another major hallmark of EMT. Expression of cytokeratin and vimentin can be used to monitor the status of EMT in breast cancer (25, 26). Additionally, mesenchymal-related cytoskeletal markers(vimentin, FSP1, α-SMA), ECM proteins (fibronectin, type I/III collagen) and matrix metalloproteinases (MMPs) were also significantly upregulated during EMT (25).

An important feature of EMT is reversibility. Mesenchymal–epithelial transition (MET) is the reverse process of EMT, and both of them contribute to the tumor metastasis (27, 28). Another notable feature of EMT is that the transition from epithelial to mesenchymal status in adult tissues is often incomplete, and few cells can complete the entire EMT process (12, 27). Therefore, most cancer cells undergoing EMT will remain in the epithelial/mesenchymal intermediate state, also known as hybrid E/M or partial EMT phenotype. Due to varying degrees of activation of the EMT program, a spectrum of partial EMT cells is generated, which is considered to have higher plasticity than complete mesenchymal cells (29).

## 3 EMT-related regulatory networks in breast cancer

**Figure 2:**
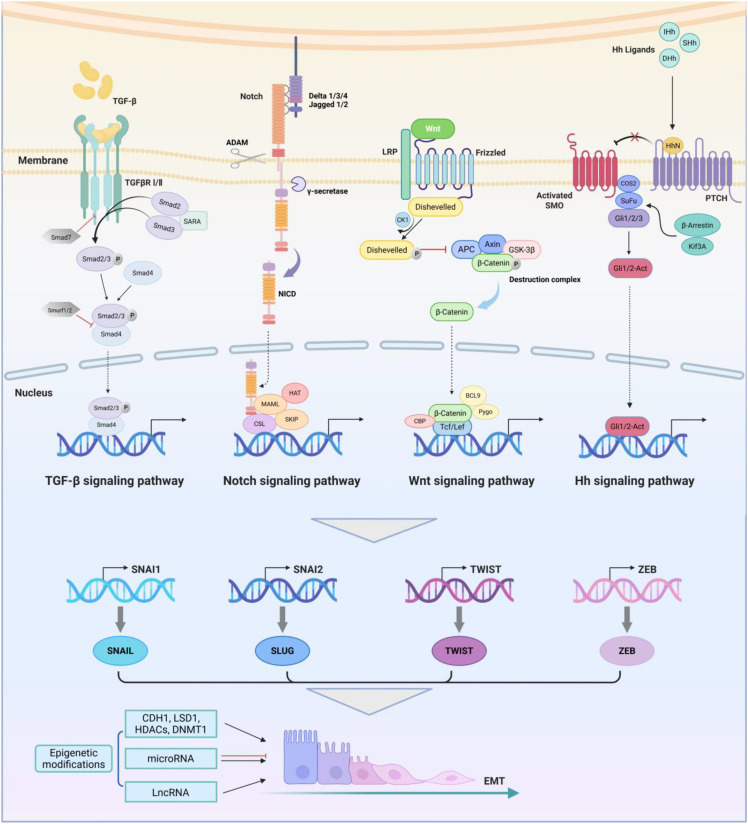
EMT-related regulatory networks in breast cancer. The TGF-β receptor is a complex composed of two types of transmembrane serine threonine kinase receptors. The activation of the canonical TGF-β signaling pathway is mediated by SMAD transcription factors. Activated TGF-β ligands bind to TβRII, inducing the recruitment, phosphorylation, and activation of TβRI, thereby phosphorylating downstream mediators SMAD2 and SMAD3, which then form the trimer with Smad4. After entering the nucleus, the trimer binds to EMT-TFs and initiates transcription. The Notch signaling pathway is mediated by four receptors (Notch-1, -2, -3, -4) and five ligands (Delta-like-1, -3, -4 and Jagged-1, -2). The Notch receptor is activated by interaction with its ligand. The gamma-secretase is responsible for cleaving intracellular C-terminal fragments of the Notch receptor and a disintegrin and metalloproteases (ADAM) is responsible for cleaving the extracellular part, leading to the release of NICD and formation of the complex with CSL. Wnt binds to the LRP5/6-Frizzled receptor to form functional receptor complex and induce recruitment and activation of Disheveled receptor, thereby inhibiting the phosphorylation and ubiquitination of β-catenin by the destruction complex. Dissociative β-catenin in the cytoplasm enters the nucleus and initiates transcription of downstream target genes. There are three types of Hh ligands, including Sonic Hedgehog (SHh), Indian Hedgehog (IHh), and Desert Hedgehog (DHh). Hedgehog protein forms a signal active N-terminal fragment HhN via self cleavage, which binds to PTCH to activate SMO. The activated SMO binds to the COS2/Sufu/Gali trimer, causing Gli to dissociate from the trimer and become the active Gli1/2-Act, which leads to upregulation of Hh target genes. The synergistic effect of these EMT-related signaling pathways collectively activates SNAIL, TWIST, ZEB and other EMT-related transcription factors. They regulate downstream transcriptional networks together with epigenetic factors, further mediating the biological effects of EMT. Image created using Bio-Render.com software.

The initiation of EMT programs is co-regulated by multiple signaling pathways. The cooperation and crosstalk of these signaling pathways ultimately stimulate the activation of EMT-transcription factors (EMT-TFs), whose transcription targets are aforementioned hallmarks of EMT. (**Figure 2**) In addition to signaling pathways, the activation of EMT is also regulated by epigenetic modifications. In summary, EMT is a multifaceted program activated by a regulatory network formed by the combined action of multiple factors.

### 3.1 EMT-related signaling pathways

EMT is regulated and affected by multiple signaling pathways, among which the critical pathways that regulate EMT initiation in breast cancer include transforming growth factor-beta (TGF-β), Notch, Wnt and Hedgehog (Hh) pathways (30). Growing evidence suggests that these signaling pathways dominate the activation of EMT, tumor development, and the occurrence of drug resistance. We summarize the process of regulating EMT by the four critical signaling pathways in breast cancer concisely, providing a theoretical basis for subsequent targeted therapy strategies.

The TGF-β pathway is one of the earliest discovered and the most extensively studied signaling cascade in the induction of EMT (31). Activated TGF-β ligands phosphorylate downstream mediator small mothers against decapentaplegics (SMADs) by binding to TGF-β receptors and form a trimer (32). After entering the nucleus, the trimer binds to EMT-TFs and initiates transcription (33). It is reported that the Notch receptor Notch-4 plays a primary role in EMT signaling in breast cancer cells and could be a potential target (34). The Noth intracellular domain (NICD) is released through a cascade of proteolytic cleavage, which forms a complex with RBP-jk (i.e. CSL) and triggers EMT-TFs (35). The Wnt signaling pathway has been gradually discovered to play an indispensable role in regulating EMT, stemness and drug resistance of various cancers, including breast cancer (36, 37). Wnt binds to Frizzled (Fzd) receptor on the cell membrane to form Disheveled (Dvl)-Fzd complexes, and inhibits β-catenin phosphorylation that originally occurred in the construction complex, allowing it to enter the nucleus and ultimately activate EMT-TFs (38). Hh binds to its receptor Patched (PTCH) to activate Smoothened (SMO) that was originally suppressed during transport and allow the Hh signaling to be transmitted downstream to glioma (GLI) family, leading to upregulation of Hh target genes, such as EMT-TFs (39).

In addition to the pathways mentioned above, there are other signals involved with EMT in breast cancer, including Nuclear factor-kappaB (NF-κB), Phosphatidylinositol 3-Kinase (PI3K)/AKT, Mitogen-Activated Protein Kinase (MAPK), Hypoxia‐Induced Factor (HIF), Epidermal Growth Factor (EGF) and so on. These pathways crosstalk and interact with each other, forming an EMT-related signal network. For example, hypoxia activated HIF-1α promotes EMT by enhancing the Notch signaling pathway (35, 40). NF-κB signal can enhance the Hh pathway, which is also influenced by TGF-β/Smad, HIF‐1α, RAS/MAPK or PI3K/AKT signaling (41–45). EGF receptors can also regulate downstream signaling molecules, including β-catenin, RAS, MAPK, and PI3K/AKT, to regulate EMT associated events (46).

### 3.2 EMT-related transcription factors

The combined effect of signaling pathways leads to the activation of EMT-TFs. The transition of cancer cells from epithelial to mesenchymal states is regulated by EMT-TFs, including SNAIL, TWIST, ZEB, FOX, SOX, PRRX, etc (47). SNAIL, TWIST, and ZEB family are currently recognized as the main regulatory factors driving the transcription pathways of EMT, which can regulate cellular characterization, such as intercellular adhesion, cell polarity, and motility (48, 49). SNAIL family (SNAI1, SNAI2, SNAI3) and basic helix loop helix (BHLH) family (TWIST1, TWIST2) regulate EMT by downregulating epithelial gene expression and upregulating mesenchymal gene expression (50, 51). The E-box elements in the E-cadherin (CDH1) promoter play a critical negative regulatory role in gene transcription (52). ZEB family of zinc finger (ZEB1, ZEB2) regulate gene sequences by binding to E-boxes, ultimately activating transcription (53). The Snail family can also induce EMT via binding to the elements and inhibiting CDH1 (54, 55). EMT-TFs typically control expression with each other and collaborate functionally on target genes to achieve the activation of the EMT program (56). For example, TWIST and SNAIL are frequently co-expressed in breast cancer, and the expression of SNAI2 (SLUG) directly depends on TWIST1 (18).

To date, all established EMT processes involve at least one member of these EMT-TFs. However, the occurrence of EMT cannot be inferred solely from the expression of EMT-TFs in cells, as these transcription factors are also involved in other cellular processes such as proliferation and apoptosis (12). Additionally, it should also be noted that the signaling pathways have more than a unidirectional effect on EMT-TFs. Although numerous reports have shown that various signaling pathways activate EMT-TFs to induce the occurrence of EMT, there are also many studies demonstrating that EMT-TFs can stimulate the transmission of signaling pathways. For example, Snail and Slug have been shown to activate TGF-β and MAPK pathways (57, 58). The positive feedback between signaling pathways and EMT seems to be a vicious cycle, which may explain why tumor progression is often so rapid and difficult to cure.

### 3.3 Epigenetic modifications

The course of EMT is related to epigenetic alterations, which are achieved by regulating the function of EMT-TFs (59). Epigenetic modifications, including DNA methylation, histone modifications and non-coding RNA regulation, have been confirmed to be largely involved in the EMT process. It is reported that CDH1 promoter metabolism plays an important role in EMT of various human tumors, including breast cancer (60, 61). EMT-TFs, such as SNAIL and ZEB, bind to E-box on CDH1 promoter, directly leading to inhibition of E-cadherin expression (62). In addition, it was discovered that demethylases play an important role in EMT (63). The histone demethylase lysine-specific demethylase 1 (LSD1) interacts with SNAIL and is recruited into the CDH1 promoter, leading to histone demethylation (64, 65). Exosomal piRNA-17560 enhances the stable expression of ZEB1 by reducing N6-methyladenosine RNA methylation, thereby inducing EMT and chemoresistance (66).

The image of non-coding RNA as a key factor that can affect the EMT process has become increasingly prominent. MicroRNAs (miRNAs) are a group of non‐coding single‐stranded small RNAs. They bind to the 3’-UTR region of downstream target mRNA through base pairing, which can cause the degradation of target mRNA or inhibit its translation, thus they are considered as the main post-transcriptional regulators of gene expression (67, 68). Research has shown that miRNAs affect the EMT process by mediating the expression of EMT-TFs. The miR-200 family is believed to inhibit the expression of ZEB, and interestingly, ZEB simultaneously suppresses the expression of the miR-200 family through a double negative feedback loop in breast cancer (69–71). Similar double negative feedback loops can also be observed between other miRNAs and EMT-TFs, including miR-203/SNAIL1, miR-129-5p/SOX4 and miR-30a/SOX4 (72–74). Mounting evidence has indicated that long non-coding RNAs (lncRNAs) contribute to malignant behaviors of breast cancer, including EMT and drug resistance (75). It is reported that LincK can regulate the expression of ZEB1 and the function of miR-200 in breast cancer (76). Similarly, lncRNA LINC00460 has been shown to promote Cancer stem cell (CSC)/EMT-like characteristics and resistance to doxorubicin by forming a positive feedback loop with c-MYC (77). Interestingly, high expression of lncRNA SNHG6 can promote EMT initiation and tamoxifen resistance in ER-positive breast cancer by inhibiting miR-101 (78). Another new study have revealed that lncRNA PTENP1 regulates EMT and drug resistance of breast cancer through isolating miR-21 by constructing a dynamic Boolean network model (79). In murine or human breast cancer, there are other examples of lncRNA-miRNA interactions like this, and some studies have referred to these pathways as lncRNA/miRNA axis, which can exert the oncogenic effect and promote drug resistance by regulating EMT process (80).

In conclusion, the elements involved in the EMT process are not independent, nor are they acting unilaterally. These factors and signaling pathways interact and entangle with each other, synergistically regulating EMT. The same factor can affect every step of the EMT process, and downstream factors can also have a reverse impact on upstream signals. New evidence suggests that the presence of TGFβ2/Smad‐Snail1/EZH2‐miRNAs loop in TNBC can maintain EMT phenotype and induce drug resistance, which is a good example of the interaction of multiple regulatory factors (81). There are countless interplays and feedback loops like this between various elements. Therefore, the EMT-related regulatory network is a complex and dynamic system that deserves further research.

## 4 Mechanisms of EMT-induced drug resistance in breast cancer

**Figure 3:**
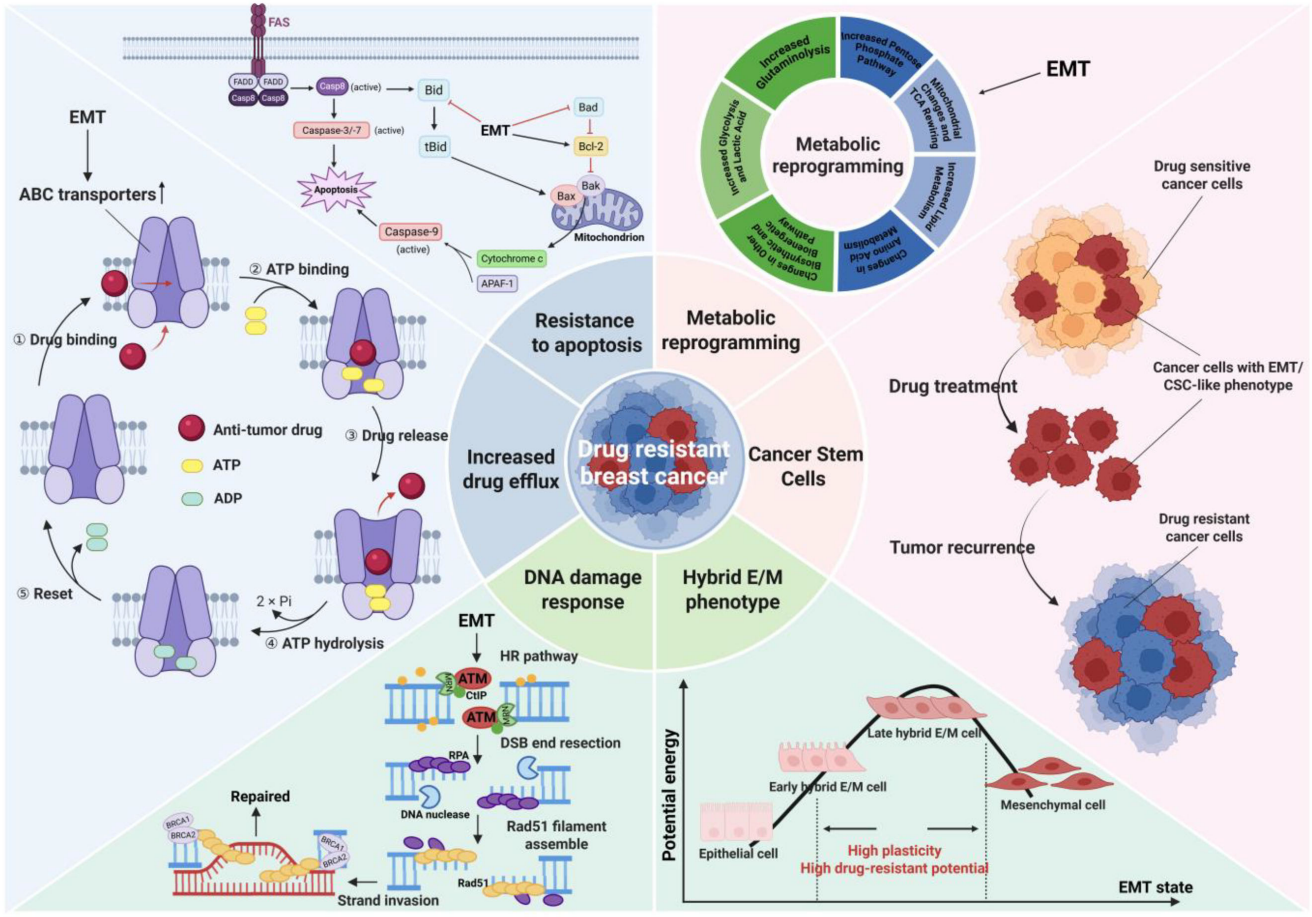
Potential mechanisms underlying EMT-induced drug resistance in breast cancer. EMT cells are highly similar in phenotype and function to CSCs, and EMT process is recognized to promote the production of CSCs. After killing drug sensitive cancer cells, residual cancer cells with EMT/CSC-like phenotype may trigger tumor recurrence and develop drug resistance. Most cancer cells that undergo EMT will remain in the hybrid E/M state, which exhibits the most prominent phenotypic heterogeneity and plasticity, enabling better adaptation to the microenvironment and resistance to drug action. The drug and ATP molecules enter the ABC transporter and bind to corresponding binding sites, forming an outward-open conformation that causes the drug to flow out of the cell. EMT promotes the expression and activation of ABC transporters, increasing drug efflux and leading to drug resistance. Cells that receive apoptotic signals activate pro-apoptotic protein and caspase through intrinsic and extrinsic apoptotic pathways to conduct apoptosis. EMT can upregulate the anti-apoptotic protein Bcl-2 and inhibit pro-apoptotic proteins, thereby resisting drug-induced apoptosis. The HR pathway is a classic DDR pathway for repairing DNA damage such as double strand breaks. EMT activates the HR pathway (such as activating the ATM promoter) to reverse the cell damage caused by platinum-based drugs, resulting in the tumor exhibiting a drug-resistant phenotype. EMT can induce metabolic reprogramming in cancer cells, resulting in marked changes such as increased glycolysis, increased lipid metabolism, mitochondrial respiratory inhibition and so on, which in turn induce tumor drug resistance. Image created using  software.

Insufficient drug dosage during chemotherapy not only fails to eradicate cancer cells far from blood vessels, but also accelerates the EMT procedure, ultimately inducing drug resistance (82). Luo et al. identified that the molecular biomarkers of HR+/HER2 metastatic breast cancer patients resistant to standard treatment were related to EMT through single-cell RNA sequencing (83). Although there have been new advances in the study of the significance of EMT in drug resistance, the molecular mechanism is incomplete due to the lack of suitable *in vivo* models and limited human samples for comprehensive research (84). Next, we will provide a summary of several widely discussed potential mechanisms underlying EMT-induced drug resistance in breast cancer (**Figure 3**).

### 4.1 Acquisition of CSC-like characteristics

Thanks to the rapid development of research related to CSCs, researchers have gained a new understanding of the mechanism of drug resistance caused by EMT. CSCs are a small subset of malignant cells that possess self-renewal ability and tumorigenesis potential (85, 86). There are studies reporting a high degree of similarity between EMT cells and CSCs (87). EMT cells can also serve as effective seeds for both primary and metastatic tumors, making it challenging to distinguish them from CSCs in function (88). In addition, the signaling pathways related to self-renewal and maintenance of CSCs highly overlap with the pathways regulating EMT, such as Wnt, Notch and Hh signaling pathways (89, 90). Moreover, CSCs isolated from breast tissue express a number of typical EMT markers (91). Most importantly, there are publications proving that breast epithelial cells or breast cancer cells can obtain pluripotent stem cell-like phenotype (CD44^high^, CD24^low^) through EMT induction, which further indicates that EMT is closely related to the production of CSCs (91, 92). EMT-TFs have been identified as key factors involved in the stemness regulation of CSCs (87). EMT-TFs, represented by SNAIL, TWIST and SOX, can induce the emergence of breast cancer stem cells (BCSCs) exhibiting CSC-like phenotypes while driving the EMT process (91, 93–95). In summary, induction of EMT can promote the production of BCSCs, which can form mammospheres and express EMT markers. Therefore, the emergence of CSC-like phenotype will be regarded as evidence and result of the occurrence of EMT process in this review.

It is generally believed that CSCs are inherently resistant to anti-tumor drugs and cause the relapse, or they can acquire resistance under the influence of the tumor microenvironment (96). It has been found that cells with CSC/EMT-like characteristics in breast cancer are resistant to neoadjuvant chemotherapy (97). Further research shows that breast cancer cells with CSC/EMT-like properties still survive after receiving neoadjuvant chemotherapy or pharmacological inhibition targeting HER2, which underscore that these cells encode drug resistance (97, 98). Therefore, cancer cells treated with anti-tumor drugs may achieve drug resistance by undergoing EMT to obtain CSC-like features (99). It may be one of the possible mechanisms by which EMT induces drug resistance.

### 4.2 Enhanced plasticity of hybrid epithelial/mesenchymal phenotype

As mentioned above, most cancer cells that undergo EMT will eventually maintain a hybrid epithelial/mesenchymal (or partial EMT) phenotype, which is also one of the reasons why EMT tends to cause drug resistance. After 8 days of TGF-β induction treatment of human mammary epithelial cells, single-cell RNA sequencing analysis was used to identify ten different EMT subgroups, most of which were in the partial EMT state rather than the complete epithelial or mesenchymal state (100). More importantly, the hybrid E/M cells possess higher metastatic and drug-resistant potential in breast cancer as compared to cells on either end of the EMT spectrum (15, 101). This is because during the entire process of epithelial mesenchymal transition along the E-to-M spectrum, individual cells generate extensive phenotypic heterogeneity, and cells in the hybrid E/M state exhibit high plasticity, which provide greater adaptability and resistance for cancer cells (12). Consistently, through multi-modal translational data-bulk, single-cell, and spatial transcriptomics, it can be concluded that breast cancer cells can obtain higher heterogeneity along EMT spectrum, thus limiting the drug efficacy (102). EMT-driven cell plasticity makes breast cancer cells resistant to paclitaxel by promoting the formation of primary cilia (103). In stage III breast cancer patients, EMT and the accompanying epithelial-mesenchymal heterogeneity serve as prognostic indicators for survival outcomes (104). In summary, most cancer cells that undergo the EMT process will finally remain in a highly plastic E/M intermediate state and acquire the ability to resist the effects of chemotherapy drugs and adapt to this microenvironment, exhibiting a drug-resistant phenotype in clinical practice. However, it is worth noting that the latest evidence claims that higher plasticity may not be directly related to partial EMT state, which is a direction that needs further exploration in the future (105).

### 4.3 Increased drug efflux and reduced drug intake

Like other types of drug resistance, an important mechanism by which EMT induces drug resistance is through the regulation of the ATP binding cassette (ABC) transporter family of proteins, which increases drug efflux and reduces drug efficacy in cancer cells (106). Currently, there are up to 16 ABC transporters in the ABC transporter family related to multidrug resistance (MDR), including P-glycoprotein (P-gp, also known as ABCB1 or MDR1), Multi-drug Resistance associated Protein-1 (MRP-1; also known as ABCC1) and Breast Cancer Resistance Protein (BCRP) (107). They actively efflux a series of commonly used anti-tumor drugs, including mitoxantrone, anthracyclines, vinca alkaloids, taxanes and other drugs suitable for breast cancer treatment (107). It is reported that EMT induction upregulates the expression of ABC transporters and exacerbates drug resistance in breast cancer (108). It has been proven that EMT-TFs are the regulators in directly modulating ABC transporters, and promoters of ABC transporter genes contain the binding sites for EMT-TFs, such as TWIST, SNAIL and FOXC2 (108). Taken together, EMT-TFs can augment the activity of ABC transporters in various breast cancer cell lines by directly binding to their promoters, thereby leading to enhanced drug efflux, which constitutes a pivotal molecular mechanism of EMT-induced drug resistance.

### 4.4 Insensitivity and resistance to apoptosis mechanisms

In addition to increased drug efflux, avoidance of drug-induced apoptosis and necrosis is another possible mechanism of EMT-induced drug resistance (109). Growing evidence suggests that EMT can induce cell apoptosis resistance by upregulating the anti-apoptotic protein Bcl-2, downregulating pro-apoptotic proteins (such as Bad, Bax, Bim, p53, Noxa), activating PI3-K/Akt pathways, or interfering with cell cycle (110–112). EMT-TFs, especially SNAIL family, play a master role in increasing resistance to apoptosis. SNAIL stimulates the PI3-K/Akt pathway and inhibits pro-apoptotic protein Bad by inhibiting PTEN transcription, thereby promoting apoptosis resistance (113). SNAIL has also been reported to interfere with the function of pro-apoptotic proteins p53 to prevent cell apoptosis and lead to drug resistance (114). Moreover, SNAIL can inhibit the transcription of cyclin D2 and block cell cycle progression, making cells resistant to apoptosis (115). In addition to activating the PI3K/Akt pathway, TWIST also promotes EMT and upregulates anti-apoptotic protein Bcl-2 to contribute to apoptosis resistance (116, 117).

EMT can also protect cells from drug-induced apoptosis by activating autophagy. The EMT-related signaling pathways, including TGF-β, RAS, WNT, and NF-κB, not only activate EMT, but also closely associate with autophagy (118). EMT-related signaling triggers, such as TGF-β and hypoxia, can effectively induce autophagy under different environmental conditions (119, 120). TGF-β induces autophagosome formation and upregulates the expression of autophagy-related genes in MDA-MB-231 cells (121). Although autophagy and apoptosis are activated by multiple overlapping upstream signals, they mostly cross-regulate each other in an inhibitory manner. Therefore, EMT-induced autophagy reduces the tendency of cells to undergo apoptosis, which manifests as drug resistance in clinical practice (122). It is reported that autophagy induces resistance of breast cells to epirubicin and pacitaxel (123–125). In summary, EMT can combat drug-induced apoptosis of mammary carcinoma cells through various pathways, leading to drug resistance.

### 4.5 Activation of DNA damage response pathways

DNA damage response/repair (DDR) is a mechanism within cells to resist DNA damage induced by external or internal factors, which monitors and transmits damage signals and makes appropriate responses (126, 127). Homologous recombination (HR) pathway is a classic DDR pathway used to repair DNA damage like double-strand break (128, 129). Some chemotherapeutic agents exert anti-tumor effects by damaging the nuclear or mitochondrial DNA of cancer cells, driving their direct or indirect death (130). For example, cisplatin is an efficient DNA-damaging agent with significant anti-cancer effects. However, some cancer cells can reverse the damage induced by anti-tumor drugs, including DNA-damaging agents, by enhancing their ability of DNA damage repair, thus exhibiting a drug-resistant phenotype (5). The resistance of platinum-based drugs is closely related to DDR. Nucleotide exit repair and HR pathway are the main DDR pathways for reversing platinum damage in cancer cells (131, 132).

A pioneering report reveals the connection between EMT and DDR. ZEB1 has been identified as a response target for Ataxia‐telangiectasia‐mutated (ATM), which is a key protein kinase in HR signaling (133). Furthermore, phosphorylated ZEB1 can interact with USP7 deubiquitylating enzyme and promote HR-dependent DDR pathways. In breast cancer, ZEB1 can also activate the ATM promoter by binding to p300/pCAFj, forming a positive feedback loop that promotes DNA repair and resists DNA damage caused by chemotherapy (134). In addition, ubiquitinated TWIST1 can modulate DDR pathway and upregulate HR gene expression (135). In summary, EMT-TFs, especially ZEB1, can promote DDR and increase HR pathway activity. As mentioned earlier, HR pathway is the main DNA repair mechanism for reversing platinum damage, which may explain why EMT promotes tumor resistance to platinum-based drugs.

### 4.6 Other mechanisms

There is mutual influence between tumor microenvironment (TME) and EMT. The stromal cells that constitute TME, including cancer-associated fibroblasts (CAFs), tumor-associated macrophages (TAMs) and T lymphocytes, interact with neighboring cancer cells by secreting cytokines or other means, activating their EMT program (30). Carcinoma cells undergoing EMT also have an impact on various cells in TME, thereby affecting tumor progression and drug resistance (30, 96). EMT cells exert immunosuppressive effects to regulate immune cells in TME. For example, when MCF-7 cells overexpress SNAIL, the function of co-cultured T cells is severely reduced (136). In addition, the EMT cells secrete or indirectly activate immunosuppressive factors, such as TGF-β, TNF and CCL5, which affect the activity of various immune cells in TME (137, 138). Spatial colorization analysis reveals that at the tumor boundary characterized by EMT, CAFs and M2-like TAMs interact to promote immune exclusion and drug resistance (139). A new research has developed a biomimetic codelivery system that can reverse the EMT and CSC-like characteristics of TNBC cells to reshape the immunosuppressive microenvironment, thereby enhancing the sensitivity of TNBC to paclitaxel (140). Therefore, EMT-induced immune reconstitution may be a possible mechanism of drug resistance in breast cancer.

Metabolic reprogramming is another potential mechanism of EMT-induced drug resistance. It is reported that overexpression of SNAIL can induce aberrant glucose metabolism in cancer cells, including increased glucose uptake and lactate production, decreased oxygen consumption by mitochondria, and so on, thus altering TME and enhancing chemoresistance (141–143). The rate of glycolysis in EMT cells is significantly increased, resulting in the production of more ATP to meet the energy requirements for wound repair and resistance to attacks, which is manifested as refractory breast cancer (144). Meanwhile, acidification of TME can induce breast cancer cells to become resistant to mitoxantrone (145). Growing evidence shows that the reprogramming of lipid metabolism is conducive to the development of drug resistance in breast cancer (146). Fatty acid synthase (FASN), as a kind of metabolic oncogene involved in neoplastic lipogenesis, has been found to induce drug resistance (147). EMT was also found to be involved in this process. New evidence indicates that CD36-mediated fatty acid uptake makes HER2-positive breast cancer cells obtain drug resistance by regulating the EMT-like phenotype (148). EMT, accompanied by changes in lipid metabolism, limits the entry of drugs into cells through the plasma membrane and prevents drug accumulation (149, 150). In addition, the occurrence of EMT also causes the upregulation of P-gp, which can remove the lipid peroxidation products induced by the application of doxorubicin, thus leading to doxorubicin resistance in breast cancer (151).

EMT-induced drug resistance is usually multifactorial and complex, resulting from the combined effects of various cells and molecules. The above only summarizes the relatively important mechanisms mentioned in recent research, and more influencing factors and comprehensive mechanisms need to be explored in the future.

## 5 Therapeutic strategies by targeting EMT program

**Figure 4:**
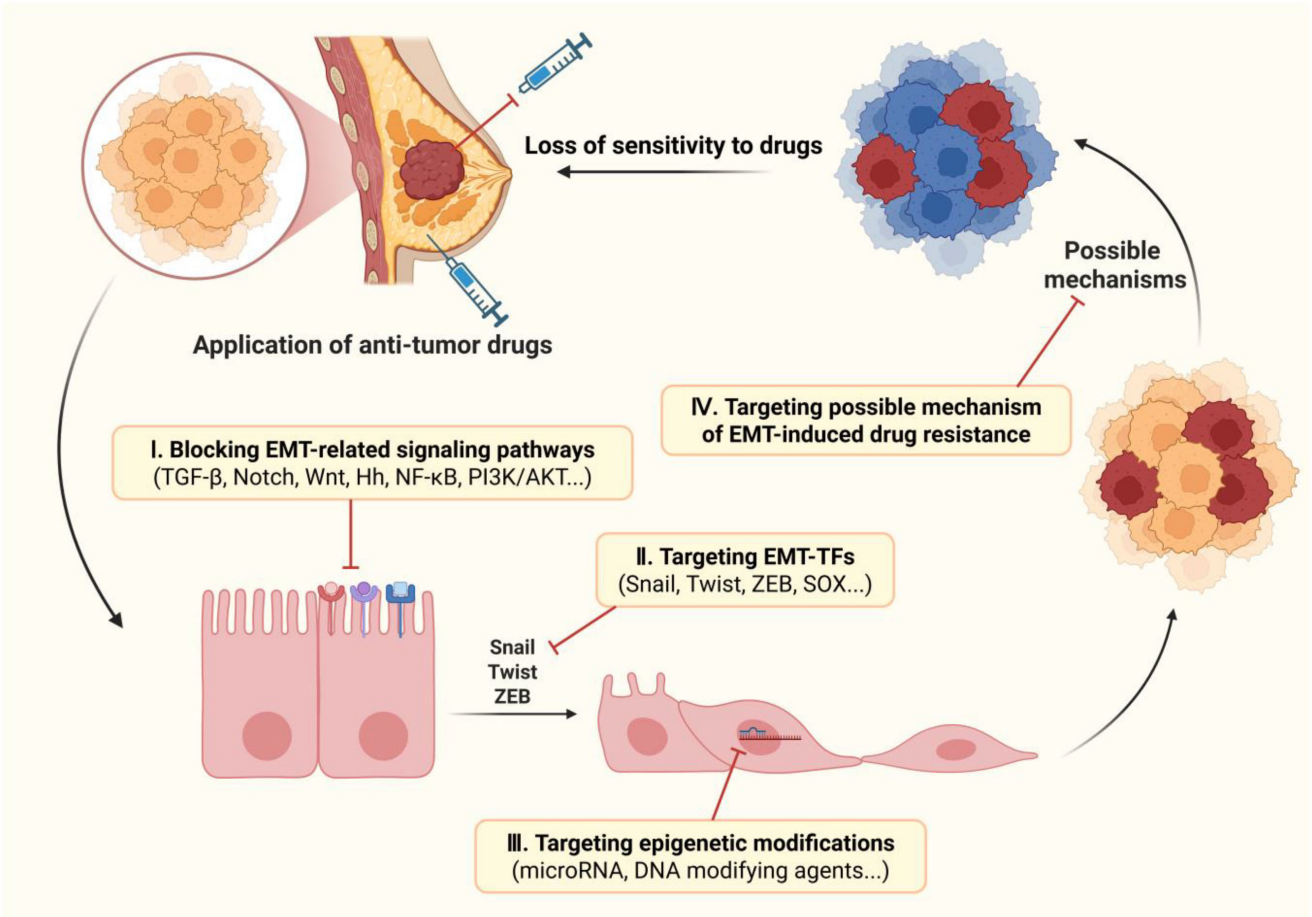
Therapeutic strategies to overcome EMT-induced drug resistance in breast cancer. There are four possible strategies for targeting EMT to overcome drug resistance in breast cancer: 1) inhibiting the occurrence of EMT by blocking the upstream signaling pathways; 2) blocking transcription of EMT by targeting EMT-TFs; 3) inhibiting EMT progression by regulating post-transcriptional epigenetic modifications; 4) maintaining sensitivity to drugs by targeting the possible mechanisms of EMT-induced drug resistance. Image created using Bio-Render.com software.

The drug resistance is not only an important focus in the development of traditional chemotherapy drugs, but also a focal point worthy of attention in emerging targeted therapies. Since EMT has been established as a fundamental mechanism that endows breast cancer cells with drug resistance and CSC-like traits, targeting EMT to control drug resistance represents a promising therapeutic strategy for breast cancer (152). In recent years, numerous studies have delved into promoting the drug sensitivity of breast cancer by blocking the EMT process and reversing the CSC/EMT-like phenotype of tumor cells. Blocking the occurrence of EMT involves disrupting the regulatory network associated with EMT, which prevents the initiation of EMT program. For example, the latest evidence shows that pentagalloyl glucose can reverse the resistance of breast cancer to doxorubicin by targeting EMT and the expression of miRNAs (153). Targeting EMT includes blocking upstream signaling pathways, directly targeting EMT-TFs, regulating epigenetic modifications (especially non-coding RNAs), and blocking the possible mechanisms of EMT-induced drug resistance. (**Figure 4**) Among them, some agents in the last strategy directly target key factors in the mechanism of resistance, such as ABC transporter family and Bcl-2, rather than overcoming drug resistance by affecting the EMT process, thus they are not included in the discussion here. Some of these molecules targeting the EMT process, especially signaling pathways, are already in the clinical trial phase and have great potential for application in future clinical treatments (**Table 1**).

**Table 1:** The molecules that have undergone/completed clinical trials and their NCT numbers of clinical projects. All information is sourced from ClinicalTrials.gov.

Name	Direct/indirect targets	NCT number
LY2157299 (Galunisertib)	TGF-β pathway	NCT02672475
PF-03084014	Gamma-secretase, Notch pathway	NCT01876251 NCT02299635
MK-0752		NCT00106145 NCT01295632 NCT00645333 NCT00756717
RO4929097		NCT01238133 NCT01071564 NCT01151449 NCT01131234
LY3039478 (Crenigacestat)		NCT02784795
LGK974	Wnt pathway	NCT01351103
OMP-18R5 (Vanicttumab)		NCT01973309
Foxy-5		NCT02020291
GDC-0449 (Vismodegib)	Hh pathway	NCT02694224
LDE225 (Sonidegib)		NCT02027376
BYL719 (Alpelisib)	PI3K	NCT05853432 NCT04762979 NCT04216472 NCT05038735 NCT02038010 NCT01870505 NCT02379247 NCT01300962 NCT02734615 NCT04208178 NCT01923168 NCT05063786
MK2206	AKT	NCT01263145 NCT01245205 NCT01344031 NCT01705340
TAK-228 (INK128, MLN0128, Sapanisertib)	TORC1/2	NCT03193853 NCT02988986 NCT02756364 NCT02049957
Curcumin	multiple signaling cascades	NCT00852332 NCT03072992 NCT01740323
LBH589 (Panobinostat)	ZEB	NCT00567879 NCT01105312 NCT00788931 NCT00632489

### 5.1 Inhibitors of EMT-related signaling pathways

#### 5.1.1 TGF-β signaling pathway

Insufficient chemotherapy may induce the initiation of EMT program by activating TGF-β signaling (154). Doxorubicin, cisplatin, paclitaxel and other anti-tumor drugs have been shown to induce the expression of TGF-β1 and the occurrence of EMT in various malignancies (154–156). For example, it has been reported that MDA-MB-231 cells treated with cisplatin have elevated levels of TGF-β, making themselves resistant to the cytotoxic effects of cisplatin (157). Another research shows that application of epirubicin can activate the TGF-β pathway in TNBC cells and regulates EMT-related markers, ultimately leading to drug resistance (158). TGF-β induces breast cancer cells to transform into partial EMT phenotype and exhibit CSC-like characteristics, which may explain why TGF-β can induce drug resistance through EMT (100). More and more similar studies have focused on the relationship between TGF-β signaling pathway and drug resistance, reminding us that the pathway may be a therapeutic target for drug resistance in breast cancer.

There is a study reporting that neutrophil extracellular traps activate the TGF-β signaling pathway by inducing SMAD2 phosphorylation in breast cancer, leading to EMT and chemotherapy resistance (159). However, TGF-β type I receptor inhibitor (TβRI), which inhibits SMAD2 phosphorylation, can block the activation of TGF-β signaling and reduce the expression of EMT-related genes, thereby improving and reversing the resistance response caused by chemotherapy (159). Another research has also reported that chemotherapy drugs lead to drug resistance through inducing the occurrence of EMT, however, TβRI kinase inhibitor (TβRI-KI) can reverse the EMT program and combined treatment with doxorubicin may improve efficacy and reduce the dosage of doxorubicin (155). Similarly, TβRI-KI LY2157299 (Galunisertib) can block the conduction of the TGF-β pathway and inhibit the development of drug-resistant CSCs and tumor recurrence induced by paclitaxel (160). TβRI/TβRII inhibitor LY2109761 can also reverse EMT and enhance chemosensitivity by inhibiting the TGF-β signaling pathway (161, 162). Curcumin, as a natural agent, is closely related to the occurrence and development of cancer (163). Curcumin can suppress doxorubicin-induced EMT and improve the efficacy of chemotherapy by inhibiting TGF-β/Smad and PI3K/AKT signaling cascades (164).

In addition to the aforementioned, there are also many TGF-β inhibitors that have been provided with compelling ability to block the TGF-β pathway and reverse EMT, including Ki26894, LY364947, IN-1130, SM16, SB-431542, YR-290 etc (165–170). Unfortunately, TGF-β pathway is more considered to be related to tumor growth and metastasis, so these inhibitors are mostly used to control breast cancer growth and metastasis, rather than reduce drug resistance. Thus, whether they can also reverse drug resistance necessitates more comprehensive and in-depth investigation.

#### 5.1.2 Notch signaling pathway

The importance of the Notch signaling pathway in EMT program and drug resistance has been recognized, and regulating the Notch pathway may be a good approach to overcome drug resistance. The latest report has shown that imatinib, a tyrosine kinase inhibitor, has been proven to have the ability to reverse EMT by inhibiting the Notch pathway and significantly reduce the stemness of TNBC cells and induce apoptosis (171). In the past few decades, many methods have been developed and reported to regulate cancer drug resistance by inhibiting the activation of the Notch pathway, including gamma-secretase inhibitors (GSIs), ADAM inhibitors, monoclonal antibodies, etc (172). Among them, the development prospect of GSIs in reversing drug resistance is gratifying. GSIs, as the first proposed and successfully developed Notch pathway inhibitors, have shown potential in increasing tumor sensitivity to chemotherapy, whereas GSIs combined with conventional therapy have been reported to have better efficacy than using GSIs alone (173). The mechanism of GSIs reversing drug resistance in breast cancer is multifactorial. GSIs block the Notch signaling pathway by inhibiting gamma-secretase, thereby regulating CSCs, EMT, ABC transporters, and affecting crosstalk between the Notch pathway and other pathways (172). GSIs have been reported to be able to reverse drug resistance in breast cancer through the above mechanisms and achieve better therapeutic effects when combined with conventional drugs in clinical trials.

Doxorubicin can induce overexpression of MRP-1, an ABC transporter, in breast cancer cells by activating Notch pathway (174). DAPT, as a GSI, can reverse this process, reducing doxorubicin efflux and enhancing doxorubicin-induced apoptosis program. High expression of Nicastrin and Notch4 in breast cancer cells can induce the acquisition of EMT phenotype and tamoxifen resistance. Anti-Nicastrin mAbs and GSI PF03084014 can inhibit the expression of EMT-related molecules and partially alleviate drug resistance (175). Docetaxel induces the initiation of EMT program and drug resistance of breast cancer cells by activating Notch pathway, and PF-03084014 combined with docetaxel can reverse the above process (176). The combination therapy of MK-0752 and docetaxel can reduce BCSCs in breast tumor transplants by inhibiting the Notch pathway and enhance the efficacy of docetaxel (177). In addition, biopsy results of clinical trials showed a reduction in CSC-like phenotype cells and a decreased mammosphere after the combined therapy, which can be seen as a reversal of EMT. A classic GSI, RO4929097, has been proven safe and effective when used in combination with other conventional drugs in various clinical and preclinical trials (178, 179). However, there are limited reports on whether RO4929097 can reverse EMT and drug resistance by inhibiting the Notch pathway. It is reported that short-term treatment of breast cancer with tamoxifen or fulvestrant will increase the activity of BCSCs by upregulating Noth4 target gene, while RO4929097 can inhibit BCSCs and alleviate drug resistance (180). Another Notch inhibitor, LY3039478 (Crenigacestat) has been reported to have poor tolerability and results in disappointing clinical survival rates for breast cancer patients (181).

#### 5.1.3 Wnt signaling pathway

Wnt signaling pathway plays a role in promoting tamoxifen acquired drug resistance in breast cancer. Won et al. have reported that the tamoxifen-resistant MCF-7 cell line possesses mesenchymal phenotype and significantly increased level of β-catenin (182). After treatment with classical WNT inhibitor ICG-001 or β-catenin siRNA, the expression of active β-catenin was inhibited, and viability of the drug-resistant cell line was also reduced (182). Another Wnt inhibitor, IWP-2, has also been proved to improve the sensitivity of breast cancer cells to tamoxifen by inhibiting EMT (183). Similarly, pyridine derivatives reverse the resistance of MCF-7 breast cancer cells to tamoxifen by inhibiting the activation of Wnt/β-catenin and NF-κB pathways (184). It can be inferred that the Wnt signaling pathway may act as a potential therapeutic regimen for alleviating resistance.

After treating TNBC cell lines with Wnt inhibitor FH535, the expression of EMT-related markers (E-cadherin) and EMT-TFs (Snail and Twist1) was significantly downregulated, indicating partial reversal of the EMT process (185). Activation of Wnt/β-catenin pathway will induce trastuzumab resistance in breast cancer cells with HER2 overexpression, thus, knocking out Wnt3 by siRNA can result in downregulation of EMT-related expression and restoration of trastuzumab’s inhibitory effect on cell growth (186). Porcupine is a key factor regulating the release of Wnt ligands, and LGK974 is a specific inhibitor of it (187). It is reported that enhanced activity of the Wnt/β-catenin pathway induces drug resistance in TBNC cells and enhances the expression of CSC-like markers, which can be reversed by LGK974 (188, 189).

As a key receptor for Wnt/β-catenin signaling, Frizzled-7 (Fzd7) is abnormally expressed in TNBC, which is associated with poor prognosis and resistance to chemotherapy (190, 191). Fzd2 promotes the maintenance of mesenchymal phenotype in breast cancer cells, and endows cells with stemness and drug resistance by combining Wnt5a/b and Wnt3 (192). Knockout of Fzd2 significantly reduced the expression of ABC transporter subfamily G isoform 2 (ABCG2) and IC50 of paclitaxel, indicating that knockout of Fzd2 enhanced the sensitivity of breast cancer cells to paclitaxel. Bevacizumab, as a first-line combination drug for various cancers, has limited therapeutic effect on TNBC, because it simultaneously activates Wnt/β-catenin signaling to induce EMT process and stemness of breast cancer cells (190, 193, 194). A novel humanized antibody, SHH002-hu1, can specifically target Fzd7-positive cells and block the Wnt/β-catenin pathway, thereby inhibiting EMT and enhancing the anti-breast cancer effect of bevacizumab (195). Another Fzd receptor inhibitor OMP-18R5 (Vanicttumab), which has entered the clinical trial phase, combined with paclitaxel has more excellent anti-tumor effect than paclitaxel alone in the treatment of breast cancer (194).

Salinomycin has been proved to be an inhibitor of Wnt/β-catenin signaling pathway in breast cancer (196). A study first identified that salinomycin had specific toxicity against BCSCs and reversed the general resistance of breast cancer to multiple drug therapies, but the mechanism by which salinomycin inhibited CSCs was not explained in this research (197). Over a decade later, another study confirms that the combination of salinomycin and doxorubicin can reverse the resistance of adriamycin-resistant breast cancer cells (198). In addition, the genes related to the Wnt/β-catenin pathway and EMT was found to be downregulated, which suggested that this combination therapy can suppress CSCs by inhibiting the Wnt pathway and inverting the EMT process, ultimately reversing the drug resistance of breast cancer cells. Salinomycin also can remove markers on the surface of breast cancer cells by inhibiting Wnt signaling transduction, such as CD44 and ABCG2, a drug resistance marker (199).

The role of Wnt signaling pathway in breast cancer has been very clear and inhibitors of Wnt pathways are constantly being developed and entering clinical trials (199). Endogenous agents and pharmacological inhibitors targeting various elements of Wnt pathway have also been widely studied and reported, such as Foxy-5 (Wnt5a mimetic), DKK3, ZFP57 and so on (200). However, these Wnt inhibitors are mainly reported to prevent cell proliferation and tumor metastasis by inhibiting Wnt signaling. It is unclear whether they can improve the drug sensitivity of breast cancer by reversing the Wnt/EMT/drug resistance axis, which is also the direction we can explore and study in the future.

#### 5.1.4 Hh signaling pathway

Overexpression of Hh pathway has been proved to regulate the proliferation and self-renewal of CSCs in different cancers, including breast cancer, and can induce chemotherapy resistance by activating multiple pathways (201). Analysis of embryonic pathways showed that EMT markers were significantly increased in TNBC cells resistant to paclitaxel and doxorubicin, accompanied by activation of the Hh pathway and Notch receptor expression (202). Moreover, cilengitide is reported to overcome the resistance of HER2-positive breast cancer to trastuzumab by targeting ITGβ3 to inhibit the activity of Hh pathway and the transcription of EMT-TFs (203). Therefore, targeting Hh signaling pathway is a potential therapeutic direction to alleviate chemotherapy resistance and improve prognosis of breast cancer.

Cyclopamine (11deoxojervine) is the prototype of Hh inhibitor and is currently widely studied and used as an agent for preclinical studies (204). It achieves the effect of blocking the Hh signaling by binding to SMO signaling elements and inactivating them. Nitidine chloride (NC), a natural bioactive alkaloid, has been proved to have anti-cancer effect and enhance the inhibitory effect of doxorubicin on breast cancer (205). NC regulates the expression of EMT-related markers and reverses EMT by inhibiting Hh pathway, while reducing the CSC/EMT-like properties of breast cancer cells by regulating the pathway (206). Moreover, the combination of cycloamine and NC can enhance the above effect, which indicates that cycloamine can also enhance the sensitivity of breast cancer to anti-cancer drugs by inhibiting Hh pathway (206). The consistent research results show that another non-canonical Hh inhibitor GANT61 (Gli1 inhibitor) can effectively increase the expression of E-cadherin in breast cancer cells and down-regulate CSC/EMT-like phenotype, thereby promoting cells apoptosis (207–209). Moreover, its combination therapy with paclitaxel enhances the efficacy of chemotherapy drugs for anti-cell growth and anti-CSC activities (207). Some SMO inhibitors, such as IPI-926, GDC-0449 (Vismodegib) and LDE225 (Sonidegib), have undergone clinical trials for breast cancer treatment (210–212). TNBC cells treated with GDC-0449 or LDE225 showed downregulation of both Hh target genes and genes regulating CSC-like phenotype (212). Furthermore, the combination of LDE225 and docetaxel can improve the sensitivity of tumors to chemotherapy, and its safety and efficacy have been demonstrated (211, 212).

#### 5.1.5 Other signaling pathways

**Figure 5:**
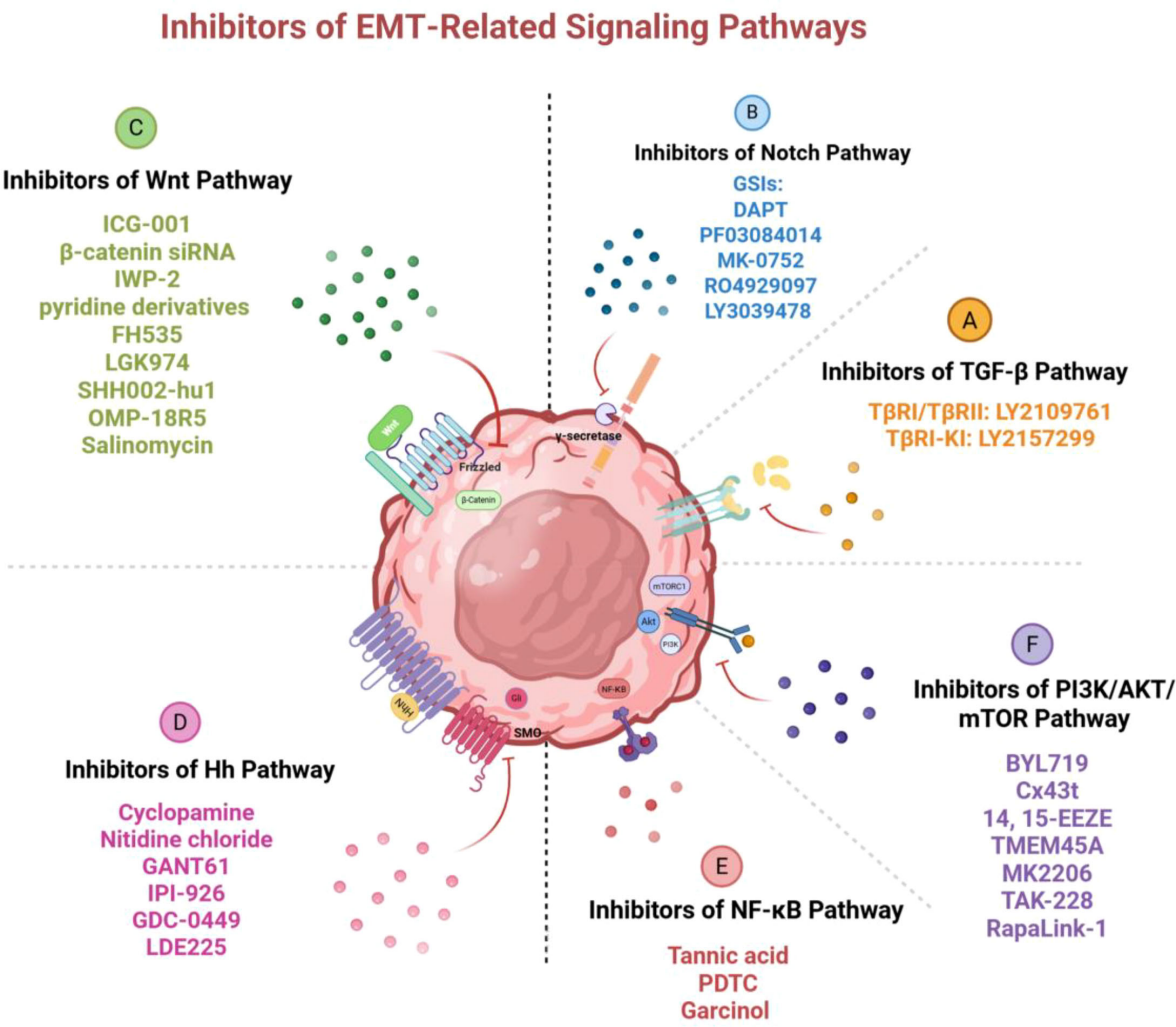
Inhibitors of EMT-related signaling pathways. Various inhibitory molecules block the activation of these pathways and the occurrence of EMT by targeting key factors in the signaling pathways, thus reversing drug resistance in breast cancer. Image created using Bio-Render.com software.

Although there are many signaling pathways proven to be associated with EMT or drug resistance, the exact description of signaling pathways that can induce drug resistance through EMT is limited, and there are relatively few reports on corresponding inhibitors (**Figure 5**). Among them, there are comparatively more discussions on the NF-κB and PI3K/AKT/mTOR signaling pathways. Tannic acid can inhibit the activation of EMT and NF-κB signaling pathway in MCF-7 cells, thereby suppressing the formation of drug-resistant BCSCs and the expression of stemness markers (213). Similarly, the co-delivery system of NF-κB inhibitor PDTC and doxorubicin can alleviate the multidrug resistance of breast cancer (214). Garcinol has been reported to inhibit NF-κB/Twist1 signaling activated by paclitaxel and downregulate the expression level of EMT-TFs, thus significantly improving the efficacy of paclitaxel in breast cancer in the orthotopic breast cancer model (215). Recently, an increasing number of PI3K inhibitors have entered the clinical trial stage and been proven to be effective in combination with other traditional chemotherapy drugs for advanced breast cancer (216). Among them, the isoform-specific PI3K inhibitor BYL719 (Alpelisib) has been shown to overcome eribulin resistance by inhibiting EMT and stemness of BCSCs (217). In addition to classic PI3K inhibitors, Cx43t is also proved to inhibit the activation of PI3K/Akt signaling by reducing Akt phosphorylation, thereby suppressing EMT and increasing tamoxifen sensitivity (218). Luo et al. reported that 14, 15-EET induces the occurrence of EMT and cisplatin resistance by activating the FAK/PI3K/AKT pathway, thus the antagonist of 14, 15-EET, 14, 15-EEZE, can reverse EMT and cisplatin resistance in breast cancer (219). mTOR has been reported to interact with EMT-related signaling pathways, including PI3K/Akt, Notch, TGF-β, and non-coding RNAs to maintain BCSC-like characteristics of cancer cells and mediate drug resistance (220). The transmembrane protein-45A (TMEM45A) induces glycolysis and EMT program by activating AKT/mTOR signaling pathway, therefore the siRNA targeting TMEM45A can reverse the above pathway and improve the sensitivity of breast cancer to palbocilib (221). MK2206 (AKT kinase inhibitor), TAK-228 (formerly INK128 or MLN0128, dual TORC1/2 inhibitor) and RapaLink-1 (mTOR inhibitor) can alleviate drug resistance by regulating the stability of CSCs phenotype and inhibiting cell viability (222). Stevens LE et al. found that pSTAT3 regulates EMT-related genes in inflammatory breast cancer cell lines resistant to paclitaxel and doxorubicin (223). Meanwhile, the combination therapy of paclitaxel and JAK2/STAT3 inhibitor, AZD1480, can prevent the occurrence of the drug-resistant subpopulation with EMT-like characteristics (223). DUSP4 (MKP-2) can block the initiation of EMT program by inhibiting the activation of JNK signaling pathway, and restore the sensitivity of breast cancer cells to doxorubicin (224).

Due to the crosstalk and interference between signaling pathways, which affect and interact with each other, a drug or inhibitor may induce the participation and alteration of multiple factors and signaling pathways simultaneously. For example, besides suppressing TGF-β/Smad and PI3K/AKT signaling, curcumin is also reported to be associated with multiple signaling cascades, such as Notch, NF-κB and Wnt/β-catenin pathway (163). Especially in the Wnt pathway, curcumin hampers activation of Slug and suppresses CSCs by blocking nuclear translocation of β-catenin (225). Similarly, in addition to targeting the Wnt pathway, salinomycin in combination with budesonide may suppress stemness of TNBC cells and activate apoptosis by inhibiting AKT/mTOR pathway and EMT (226). Pentadecanoic acid can inhibit multiple survival signaling pathways (MAPK, ERK1/2, mTOR and EGFR) and EMT, resulting in reversal of tamoxifen resistance (227). Consistently, BYL-719, as a PI3K inhibitor, can not only block the PI3K/AKT/mTOR pathway, but also inhibit the Notch, JAK/STAT, and MAPK/ERK pathways, ultimately inhibiting EMT and overcoming drug resistance (217). Therefore, the action of a drug or inhibitor may involve more than one signaling pathway, and overcoming drug resistance may be the result of the synergy and interaction of multiple pathways.

### 5.2 Inhibitors of EMT-TFs

Targeting EMT-TFs is another therapeutic strategy to overcome drug resistance in breast cancer. It is reported that overexpression of Snail in MCF-7 cells increases the level of P-gp and shows a tendency to develop resistance to adriamycin, therefore, Snail is a promising target (228). SNAI1 enhancer RNA depletion inhibits the EMT process and chemoresistance of breast cancer cells (229). MCF-7 cells transfected with pCDNA3.1-Snail can promote EMT characterization, which leads to an increased expression of BCRP and drug resistance to mitoxantrone (230). AMP-activated protein kinase (AMPK) agonists can enhance the sensitivity of TNBC cells to chemotherapy by phosphorylating Snail1 (231). The interaction between N-terminal SNAG repressor domain and LSD1 plays an important role in Slug activating EMT (65, 232), and LSD1 can maintain the CSC-like phenotype and induce doxorubicin resistance in breast cancer (233). The application of LSD1 inhibitors, 2-PCPA and GSK-LSD1, can significantly reduce CSCs, and the combination therapy with doxorubicin improves the sensitivity of cancer cells (233).

The overexpression of TWIST1 in palbociclib-resistant luminal breast cancer activates EMT (234). Moreover, the survival time of mice treated with pSilencer-twist and adriamycin was significantly prolonged compared to mice treated with adriamycin alone, suggesting that inhibiting Twist may be a possible method to enhance chemotherapy efficacy and reverse drug resistance (20). Similarly, Twist1 siRNA can reverse the high expression of EMT markers induced by adriamycin, and the anti-cancer efficacy in combination with adriamycin is significantly better than that of monotherapy (20). The CDK1 inhibitor RO-3306 can significantly inhibit the CSC/EMT-like phenotype and increase the sensitivity of TNBC cells to cisplatin and paclitaxel by downregulating the protein level of TWIST1 (235).

Growing evidence shows that ZEB influences the sensitivity of cancer cells to chemotherapy by regulating EMT (236). LBH589 (Panobinostat) is reported to mediate the inhibition of EMT by targeting ZEB expression, and inhibit BCSCs and enhance the apoptosis of TNBC cells by regulating EMT when combined with salinomycin (237, 238). Eribulin reverses EMT progression by disrupting the interaction between ZEB1 and SWI/SNF, thereby preventively increasing the sensitivity of TNBC cells to drugs (239). PEG10-siRNA has been reported to inhibit EMT and overcome drug resistance by activating SIAH1, the post-translational degrader of ZEB1 (240).

Although EMT-TFs usually exert effects by directly affecting EMT, there is evidence to suggest that EMT-TFs can also induce drug resistance through other pathways without affecting the EMT process. It is reported that Snail or Slug induces tamoxifen resistance in breast cancer by activating EGFR/ERK pathway independent of EMT, and inhibitors of EGFR/ERK pathway can restore the sensitivity of cancer cells with high expression of Snail/Slug to tamoxifen, without reversing the EMT phenotype of these cells (241). This further confirms what was previously mentioned, that regulation of EMT-TFs may be one of the necessary conditions for the occurrence of EMT, but EMT is not the inevitable result of abnormal expression of EMT-TFs (12). As mentioned above, EMT-TFs can further activate related signaling pathways through positive feedback to enhance EMT, so targeting EMT-TFs can also inhibit activation of signaling pathways. It is reported that after Sox4 expression of breast cancer cells is knocked down by siRNA, Wnt/β-catenin signaling is also observed to be synchronously inhibited through feedback loop, and subsequently, EMT, CSC-like features and cisplatin resistance are reversed (242).

### 5.3 MicroRNA

In recent years, many studies have delved into and reported on the relationship between miRNAs and EMT-induced drug resistance, and the roles of miRNAs are gradually clarified (243). Some miRNAs can reverse drug resistance by targeting the signaling pathways or EMT-TFs mentioned above, while others can exert their effects through other key molecules related to EMT. Herein, we only introduce the tumor suppressor miRNAs that are prominent in breast cancer and the critical processes to function. Other miRNAs are listed in **Table 2**.

**Table 2:** EMT‐associated tumor suppresser miRNAs and their targets for drug resistance in breast cancer.

microRNA	Direct/indirect targets	Reference
miR-27b	ENPP1, ABCG2 HMGB3	(244, 245)
miR-30	SOX4, TGF-β/Smad TWF1	(73, 246)
miR-34a	MCT-1 Notch/NF-κB, RAS/RAF/MEK/ERK	(247, 248)
miR-125b	Sema4C	(249)
miR-129-5p	SOX4, Twist1, Snail	(72, 250, 251)
miR-149-5p	IL-6, SNAIL	(252)
miR‐199a‐5p	PIK3CD	(253, 254)
miR‐200 family (miR-200a/b/c, miR-141, miR-429)	ZEB c-MYB FN1	(70, 255–264)
miR-205	Notch2, ZEB HOXD9, Snail	(265–267)
miR-340-5p	LGR5, Wnt/β-catenin	(268)
miR-375	HOXB3 metadherin	(269, 270)
miR-383	Gadd45g	(271, 272)
miR-452	Slug	(273)
miR-489	Smad3	(274)
miR-622	HIF-1α	(275)
miR-644a	CTBP1, p53	(276)
miR-671-5p	FOXM1	(277)
miR-708-3p	ZEB1, CDH2, vimentin	(278)
miR-873	ZEB1	(279)
miR-4521	FOXM1	(280)
miR‐6838‐5p	WNT3A	(281)

The miR-200 family, as one of the most widely studied EMT-related miRNA, consists of five members, namely miR-200a, miR-200b, miR-200c, miR-141, and miR-429 (282, 283). MiR-200 family members directly target ZEB to exert inhibitory effects and reverse EMT processes by upregulating E-cadherin expression (70, 255–258). For TNBC cells in the mesenchymal state, tamoxifen can promote upregulation of miR-200c through demethylating its promoter, thereby reversing EMT and increasing sensitivity to traditional chemotherapeutic agents (259). MiR-200c restored the sensitivity of HER2-positive breast cancer to trastuzumab by targeting ZNF217/TGF-β/ZEB1 axis and suppressing CSC-like phenotype (260, 261). MiR-200b/c regulates E-cadherin by targeting ZEB, thereby inhibiting EMT and increasing sensitivity of breast cancer cells to doxorubicin (262). The overexpression of miR-200b/c has also been found to downregulate c-MYB, thereby reversing EMT-induced tamoxifen resistance in ER-positive breast cancer (263). In addition, miR-200b can also reverse EMT-induced chemoresistance by targeting FN1 (264).

The miR-30 family is regarded as an important group of microRNAs that negatively regulates the malignant behaviors of tumors. It has been demonstrated that the downregulation of miR-30a facilitates EMT process and metastasis by modulating EMT-TFs (284–288). Moreover, miR-30a inhibit the initiation of EMT program and drug-resistant CSC-like phenotype by forming a double negative feedback loop with SOX4 (73). MiR-622 can exert the inhibitory effect of miR-30a on EMT and drug resistance by increasing its expression (275). Another family member, miR-30c, has also been reported to directly target TWF1 to reverse EMT, thereby restoring sensitivity of cells to chemotherapy (246).

The miR-34 family, especially miR-34a, is one of most intensively studied miRNAs in breast cancer (289). The combination therapy of miR-34 and traditional anti-cancer agents can inhibit drug resistance in various types of cancer (290). MiR-34a is reported to be able to target EMT-TFs to inhibit the EMT process (291). In TNBC, miR-34a targets MCT-1 to control M2 macrophages polarization, thereby reprogramming EMT and inhibiting stemness closely associated with drug resistance (247). Furthermore, the combination treatment of miR-34a and doxorubicin can significantly downregulate the expression of Snail by inhibiting the Notch/NF-κB and RAS/RAF/MEK/ERK pathways, thereby preventing doxorubicin-resistant breast cancer progression (248).

Although miR-129 may play a dual role in the development of tumors, more evidence tends to suggest that it acts as a tumor suppressor to prevent the malignant progression of breast cancer. In MCF-7 cells treated with adriamycin, the enhanced miR-129-5p expression significantly downregulates the expression of mesenchymal markers (vimentin and N-cadherin), indicating inhibition of EMT (72). By negatively regulating the expression of SOX4, miR-129-5p significantly reduces the IC50 of several drugs, including adriamycin, which proves that breast cancer cells are more sensitive to drugs. In addition, miR-129-5p negatively regulates Twist1 and Snail, therefore reverse the EMT process and eliminate epirubicin resistance (250, 251).

The role of miR-205 in different types of cancer is controversial, with dual effects of inhibition or carcinogenesis. According to existing reports, miR-205 typically exhibits tumor suppression and chemosensitivity enhancement in breast cancer, even though the specific effects are variant in different subtypes (292). MiR-205-5p has been proved to enhance the sensitivity of breast cancer to chemotherapeutic agents, including doxorubicin and docetaxel (293, 294). MiR-205 can negatively regulate the phenotype and activity of drug-resistant BCSCs by inhibiting related elements of EMT (265, 295). There are many known targets of miR-205 in breast cancer, including transcription factors ZEB1, ZEB2 and SIP1, which are responsible for regulating EMT (69, 267, 292). The upstream regulators of miR-205, polycomb protein MEL-18 and ligand Jagged1 also suppress the initiation of EMT via this pathway (267). Moreover, miR-205-5p can inhibit chemoresistance of TNBC by regulating Snail (266). The miR-205 and miR-200 family have similar functions and share several target genes, such as ZEB1 and SIP1, which may suggest that they may share more commonalities (69, 296). The combination therapy of miRNA-200/205 has been increasingly reported for regulating EMT and overcoming drug resistance (297).

The inhibitory function of some microRNAs on EMT and their anti-tumor metastasis effects have been widely reported, but whether they can overcome drug resistance by suppressing EMT has not been clearly revealed, which will be an orientation worthy of further research (298). It is worth noting that the role of microRNA in breast cancer may not be unidirectional promotion or inhibition, but often dual action. MiR-125b has been proved to reverse EMT and prevent drug resistance in many cancers, including breast cancer and lung cancer (299, 300). However, other studies reported the opposite results simultaneously that high levels of miR-125b are more likely to induce drug resistance and lead to poor prognosis in breast cancer patients (301, 302). The miRNAs listed above with anti-tumor effects in breast cancer may simultaneously promote malignant progression in other tumors or subtypes, which further reflects that the tumor environment is a complex and multifactorial system. Thus, the therapeutic strategy targeting miRNA still requires further exploration and investigation for future clinical application.

### 5.4 Limitations of molecules targeting EMT

Although some EMT inhibitors have entered clinical trials and been proven to improve the efficacy of conventional therapies, the safety in the long-term use of them is currently unclear (303). Connolly EC et al. first reported that long-term use of LY2109761 may induce an increase in the levels of EMT-related markers, such as E-cadherin, and would lead to acquired resistance to LY2109761 (304). Subsequently, there is increasing evidence that although blocking TGF-β signaling may provide clinical benefits, treatment with TGF-β inhibitors alone may lead to serious adverse reaction (156). In addition, the toxicity of EMT inhibitors remains a potential risk. For example, small molecule TGF-β inhibitors have shown severe cardiac toxicity in preclinical animal models (305, 306). miRNA is another promising research field for overcoming drug resistance. miRNAs may play opposite roles in different cancers, and even their functions may vary in different subtypes of the same cancer. This guides us to make more precise distinctions about the roles of miRNAs in tumors. In the past 20 years, the number of identified miRNAs and their targets has been increasing at an incredible rate. However, there are still many questions that need to be answered before miRNA therapy can be widely applied in clinical practice. How to prevent microRNA degradation *in vivo* and how to efficiently targeted delivery are still unresolved issues (307). Epigenetic modifications are closely related to EMT, stemness and drug resistance, which has aroused the interest of researchers in this field and has been widely discussed. In addition to miRNAs, the corresponding epi-drugs, such as DNA modifying agents, inhibitors of histone acetyltransferase (or deacetylase or methyltransferase or demethyltransferase), are also gradually emerging, trying to be applied in the treatment of breast cancer (308).

## 6 Conclusion

EMT is a complex and dynamic biological program that typically occurs during embryonic development and tumor progression. It is essentially a major reprogramming involving gene expression, which can affect the macroscopic malignant development of tumors by regulating the fate and behavior of cells. EMT has been reported to induce invasion and metastasis of breast cancer, and its impact on drug resistance is also becoming clearer. Increasingly direct evidence shows that EMT-related markers are closely related to the resistance to therapy in breast cancer (309, 310). The present review summarizes the current knowledge regarding EMT-induced drug resistance in breast cancer. The EMT-related regulatory network constitutes a complex system, with TGF-β, Notch, Wnt, and Hh pathways being the most explored and clearly defined pathways in breast cancer. These signaling pathways crosstalk and interact with each other, collectively activating EMT-TFs, which target the hallmarks of EMT and initiate the EMT program. Currently, the SNAIL, TWIST, and ZEB families are widely acknowledged as the primary transcription factors driving EMT. Epigenetic modifications also play a role in the EMT process by influencing the function of EMT-TFs, with particular attention being paid to non-coding RNAs. None of the elements within the EMT-related regulatory network functions in isolation. They can synergize or antagonize with other elements, forming positive or negative feedback loops. The molecular mechanism underlying EMT-induced drug resistance in breast cancer is still unclear, yet several possible mechanisms have been extensively proposed. Among them, the close relationship and high phenotypic similarity between EMT cells and drug-resistant CSCs have garnered the most attention. Furthermore, the hybrid epithelial/mesenchymal state of EMT cells endows them with a high degree of plasticity, which makes them more prone to drug resistance. Additionally, increased drug efflux, resistance to apoptosis and activation of DDR pathways induced by EMT contribute to the decreasing responsiveness of breast cancer cells to anti-tumor drugs. The elucidation of the regulatory network related to EMT and the potential mechanisms of EMT-induced drug resistance may contribute to the design of better targeted therapies combined with conventional treatments. Reversing the EMT process by modifying TGF-β, Wnt, Notch, Hh or other signaling pathways is expected to overcome drug resistance in breast cancer. Numerous inhibitors of these signaling pathways have advanced to preclinical or clinical trial phase and have been proven to partially alleviate drug resistance by reversing EMT. Directly targeting EMT-TFs can also make breast cancer cells more susceptible to anti-tumor drugs. Furthermore, as the interplay between miRNAs and EMT becomes increasingly well-understood, it is also a promising direction of research to leverage tumor suppressor miRNAs to regulate EMT and restore the sensitivity of breast cancer to anti-cancer drugs.

In recent years, with the development of technologies such as tumor genomics, transcriptomics and proteomics, the association of EMT and tumor drug resistance have attracted great interest. In the future, interdisciplinary approaches should be adopted to EMT research to further confirm its correlation with drug resistance and clarify the dominant mechanisms. For instance, database-based bioinformatics analysis can be used to develop personalized and customized treatments to overcome tumor drug resistance. Although the mechanisms and influencing factors of drug resistance are becoming increasingly complex, new targets are emerging to offer new hope to cancer patients worldwide.
